# Elucidation of cold adaptation in *Glaciimonas* sp. PAMC28666 with special focus on trehalose biosynthesis

**DOI:** 10.3389/fmicb.2023.1280775

**Published:** 2023-10-18

**Authors:** Prasansah Shrestha, Jayram Karmacharya, So-Ra Han, Jun Hyuck Lee, Tae-Jin Oh

**Affiliations:** ^1^Department of Life Sciences and Biochemical Engineering, Graduate School, SunMoon University, Asan, Republic of Korea; ^2^Genome-Based Bio-IT Convergence Institute, Asan, Republic of Korea; ^3^Bio Big Data-Based Chungnam Smart Clean Research Leader Training Program, SunMoon University, Asan, Republic of Korea; ^4^Research Unit of Cryogenic Novel Materials, Korea Polar Research Institute, Incheon, Republic of Korea; ^5^Department of Pharmaceutical Engineering and Biotechnology, SunMoon University, Asan, Republic of Korea

**Keywords:** Antarctica, cold shock proteins, extremophiles, *Glaciimonas* sp., trehalose biosynthesis

## Abstract

*Glaciimonas* sp. PAMC28666, an extremophilic bacterium thriving in Antarctic soil and belonging to the *Oxalobacteraceae* family, represents the only complete genome of its genus available in the NCBI database. Its genome measures 5.2 Mb and comprises 4,476 genes (4,350 protein-coding and 72 non-coding). Phylogenetic analysis shows the strain PAMC28666 in a unique branch within the genus *Glaciimonas*, closely related to *Glaciimonas* alpine Cr9-12, supported by robust bootstrap values. In addition, strain PAMC28666 showed 77.08 and 23.3% ANI and DDH, respectively, with *Glaciimonas* sp. PCH181.This study focuses on how polar strain PAMC28666 responds to freeze–thaw conditions, Experimental results revealed a notable survival rate of 47.28% when subjected to a temperature of 15°C for a period of 10 days. Notably, two genes known to be responsive to cold stress, Trehalose 6-phosphate synthase (*otsA*) and Trehalose 6-phosphate phosphatase (*otsB*), exhibited increased expression levels as the temperature shifted from 25°C to 15°C. The upregulation of *otsAB* and the consequent synthesis of trehalose play pivotal roles in enhancing the cold resistance of strain PAMC28666, offering valuable insights into the correlation between trehalose production and adaptation to cold stress. Furthermore, research into this neglected cold-adapted variation, like *Glaciimonas* sp. PAMC28666, has the potential to shed light on how trehalose is produced in cold-adapted environments Additionally, there is potential to extract trehalose compounds from this strain for diverse biotechnological applications, including food and cosmetics, with ongoing research exploring its unique properties.

## Introduction

*Glaciimonas* are Gram-negative, rod shaped, and non-motile bacteria belonging to the family *Oxalobacteraceae* (Zhang et al., 2011). These bacteria can thrive in aerobic and microaerophilic conditions but cannot survive in anaerobic environments. Many strains of *Glaciimonas* have been found in glaciers (Zhang et al., 2011; Frasson et al., 2015; Margesin et al., 2016), as well as in water samples from a uranium mine (Chung et al., 2013). However, our Antarctica isolate, *Glaciimonas* sp. PAMC28666 was obtained from soil, and it is a complete genome. Microbes present in extreme environments such as Antarctica provide excellent opportunities to elucidate eco-physiological and biochemical adaptations of such life forms (Pearce, 2012). Habitats with permanently low temperatures have been successfully inhabited by a wide variety of organisms, known as psychrophiles or cold-adapted organisms (Collins and Margesin, 2019).

In extreme environments, such as those with varying temperatures, salt concentration, and limited nutrients and oxygen, the presence and diversity of different metabolic pathways become essential for colonization and survival. Bacteria exposed to low-temperature conditions undergo various physiological changes. One such alteration is the synthesis of proteins, which are believed to aid bacteria in withstanding freezing temperatures. These proteins, known as cold shock proteins (CSPs), are induced, or enhanced during cold shock. Many of the proteins induced by cold shock belong to the cold shock family and are categorized as either cold-induced proteins (CIPs) or cold acclimation proteins (CAPs) (Klein et al., 1999). During the early stages of the cold shock response, CIPs show a short induction that is followed promptly by a decrease before reaching a stable state whereas CAPs are continuously synthesized at high levels during prolonged growth at low temperatures. In cold-adapted bacteria, there are fewer certain proteins called CIPs compared to bacteria that thrive in moderate temperatures (mesophiles). Instead, they rely on other proteins known as CAPs to help them grow in cold conditions (Roberts and Inniss, 1992; Berger et al., 1996; Bylund et al., 1998). This aligns with the observation that, unlike mesophiles where CIPs are the main proteins produced when exposed to cold, psychrotropic bacteria can continue making proteins with less hindrance (Hébraud and Potier, 1999).

Trehalose, a well-researched compatible solute, is accumulated through increased cold acclimatization (Kandror et al., 2002). Genes involved in trehalose biosynthesis, such as *otsA* and *otsB*, encode trehalose-6-phosphate synthase and trehalose-6-phosphate phosphatase, respectively. These genes can be induced during cold shock and osmotic stress when entering the stationary phase, and they are important for converting glucose into trehalose (Giaever et al., 1988). Furthermore, when *otsAB* mRNA is subjected to lower temperature (16°C), it demonstrates notably prolonged stability in comparison to higher temperatures (37°C) (Kandror et al., 2002). Additionally, *otsA* mutant (*ΔotsA*) cells exhibit a substantial decrease in cell viability when exposed to 4°C, suggesting that trehalose serves as a protective mechanism to combat stress in low-temperature conditions.

In the present study, we examined the complete genome of *Glaciimonas* sp. PAMC28666, which is the only complete genome of *Glaciimonas* to be reported in the NCBI database so far. The primary focus was on identifying genes responsible for a range of metabolic functions that facilitate environmental adaptability. These functions encompass aerobic metabolism, growth at low temperatures, osmoregulation, synthesis of exopolysaccharides, and chemical breakdown. Furthermore, we conducted experiments to assess the functionality of specific cold-induced proteins in *Glaciimonas* sp. PAMC28666.

## Materials and methods

### Bacterial strain and growth conditions

*Glaciimonas* sp. PAMC28666 was obtained from the Antarctica soil sample and deposited by the Korean Polar Research Institute. Using various medial like Tryptone Soya Broth (TSB), Marine Broth (MB), and Reasoner’s 2A Broth (R2A), it was found that R2A broth medium showed the most favorable for seed culture. To initiate bacterial culture, the frozen sample was revived with a 1%(v/v) inoculum and allowed to grow for 48 h. For the preparation of bacterial suspension culture, the isolate was cultured on R2A agar medium to obtain single colony. Subsequently, these colonies were transferred to R2A broth and incubated at 15°C, 200 rpm for 48 h. Finally, the culture was preserved as 20% glycerol stocks at −80°C for later use.

### Phenotypic characterization

To establish optimal growth conditions, the bacterial isolate was inoculated in R2A broth at different temperatures (8, 15, 25, and 37) °C maintaining a constant pH of 7.0 ± 0.2. The growth progress was tracked by assessing the optical density of the broth culture at 600 nm using the spectrophotometer. Once the optimal temperature was identified, the growth performance was examined across varying pH levels (ranging from 4 to 10 with 1.0 pH unit intervals) in R2A broth. Furthermore, growth rate was monitored under different NaCl concentrations (ranging from 0.5 to 2.5 with 0.5-unit intervals) while keeping temperature and pH constant. The growth curve was generated using Microsoft Corporation’s Excel software.

### Genome sequencing, assembly, and annotation

*Glaciimonas* sp. PAMC28666 was cultured in R2A broth at 15°C. Genomic DNA was extracted using a QIAamp DNAMini Kit (Qiagen, Valenciam CA) and assessed for quantity and purity using the Aglient 2,100 Bioanalyzer (Agilent Technologies, Santa Clara, CA). The extracted DNA’s quality was confirmed through agarose gel electrophoresis. Subsequently, the genome was sequenced using PacBio Sequel single-molecule real-time (SMRT) sequencing technology from Pacific Biosciences (Menlo Park, CA). Raw sequence data underwent *de novo* assembly using the hierarchical genome-assembly process (HGAP v.4) protocol and HGAP4 assembly with SMRT analysis software (ver.2.3)^1^ from Pacific Biosciences (Chin et al., 2013). The fully assembled genome was submitted to the NCBI WGS database with accession number ASM1691735v1.

### 16S rRNA gene sequence analysis and identification of *Glaciimonas* sp. PAMC28666

The phylogenetic analysis of the 16S rRNA gene sequence of *Glaciimonas* sp. PAMC28666 and sequences from the most closely related genera were determined. This analysis was performed using the MEGAX software, as detailed in the work by Kumar et al. (2018). The alignment of sequences was carried out using ClustalW (Larkin et al., 2007). The phylogenetic tree was then constructed using maximum likelihood analysis (Rogers and Swofford, 1998). To construct the phylogenetic tree, the sequences of 16S rRNA of related type strains were retrieved from the EzBioCloud database^2^ (Yoon et al., 2017). The average nucleotide identity (ANI) values and the digital DNA–DNA hybridization (DDH) between the genome sequence of strain PAMC28666 and type strains of the genus *Collimonas* were also conducted. For ANI calculations, the ANI calculator available on EzBioCloud was used, while the Genome-to-Genome Distance Calculator by Meier-Kolthoff et al. (2013) was employed.^3^ Additionally, the G + C mol. % content of DNA was determined from the complete sequence.

### Genomic insights of *Glaciimonas* sp. PAMC28666

Further analysis was conducted and thoroughly reviewed prominent genes using RAST (Rapid Annotation using Subsystem Technology) (Aziz et al., 2008) platform to discover genes involved in *Glaciimonas* sp. PAMC28666 stress adaptation. To aid visualization, a circular map using the CGView^BETA^ comparison tool (Grant and Stothard, 2008) was also constructed. Based on previously published studies, cold shock proteins that are expressed in cold temperatures were predicted. The KEGG (Kyoto Encyclopedia of Genes and Genomes) (Kanehisa and Goto, 2000) pathway database was used to learn about a specific metabolic route, specifically the synthesis of trehalose. This approach allowed us to elucidate the intricate process of trehalose biosynthesis within *Glaciimonas* sp. PAMC28666.

### Physiological characterization and tolerance of freeze–thaw cycles

Seed culture was diluted 1:100 into 100 mL of R2A broth to examine the growth patterns of *Glaciimonas* sp. PAMC28666. Following this, the diluted culture was divided across 250 mL flasks and incubated in a shaking incubator at 15°C, 25°C, and 37°C at 200 rpm. The optical density at 600 nm (OD_600_) was measured every 24 h to monitor culture progression. It was determined that the exponential growth phase occurs when the relationship between log OD_600_ and time is linear. The Excel software was used to conduct a linear regression analysis and derive the equation of the best-fitting line in the linear function to calculate the growth rate.

To evaluate cold tolerance of strain PAMC28666, it was cultured at two distinct temperatures (15°C and 25°C) until it entered the exponential growth phase. Subsequently, cells grown at each temperature were individually suspended in a solution comprising 0.9% NaCl and 0.002% MgSO4, following the methodology outlined by Mei et al. (2016). This suspension was used to perform a freeze–thaw stress test that involved 24 h of freezing at −20°C and then 1 h of thawing at 25°C. Gradient dilutions up to 10^−6^ were created after each cycle in sterilized 0.9% NaCl containing 0.02% MgSO_4._ To count colony-forming units (CFU), serial dilutions were plated on R2A agar and incubated for 96 h at 25°C. CFUs post-stress were compared to the starting CFU value at time zero to calculate the survival rate. With the average survival rates obtained from three separate studies, this resulted in the proportion of cells that survived the stress.

### Trehalose production and assay

A medium containing 0.5 g of casamino acid hydrolysate, 0.5 g of yeast extract, and 0.5 g of peptone per liter was developed to produce trehalose (Difco™ R2A agar, Fisher scientific). The carbon source to produce trehalose in this medium was added as 2% glucose. The culture cell pellets were boiled at 95°C for 20 min to remove the trehalose from the pellets. After centrifuging the resulting mixes, the supernatant was collected. By continuing to boil the supernatant at 95°C until it lost half of its volume, the supernatant containing the extracted trehalose was concentrated. Furthermore, the trehalose extract sample was mixed with 495 mL of 50 mM sodium citrate buffer (pH 6.0) to create a total volume of 1,000 mL for the trehalose assay. Purified trehalase (Megazyme), with an enzyme concentration of 0.0009 UmL^−1^, was added in a volume of 5 μL to start the reaction. The 3,5-dinitro salicylic acid colorimetric method (DNS method) (Miller et al., 1960) was used to evaluate the degradation of trehalose to glucose molecules. In this procedure, 1,000 μL of the reacted sample and 300 μL of the DNS solution were mixed and boiled for 5 min. The mixture was allowed to cool at room temperature, and the absorbance was measured at 540 nm using a spectrophotometer. Subsequently, by measuring the absorbance of a standard solution that contained 20 nmol of glucose instead of trehalose, glucose production was ascertained. A control sample devoid of trehalose was used in the measurement to gauge the level of pre-existing glucose in the tested sample. Additionally, a blank sample without trehalose extract was included as a reference.

## Results

### Physiological characterization of *Glaciimonas* sp. PAMC28666

**Figure 1:**
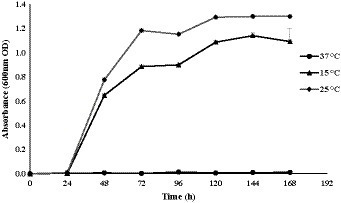
Growth curve of *Glaciimonas* sp. PAMC28666, grown at different temperatures for 168 h. Bacterial growth was evaluated by absorbance measurement at 600 nm using a spectrophotometer.

In this study, *Glaciimonas* sp. PAMC28666 was subjected to various physiological conditions, including changes in salt concentrations, pH levels, and temperatures (Supplementary Figure S1). At different temperatures (8, 15, 25, and 37) °C explored, the strain PAMC28666 did not show growth at 8ᴼC and 37ᴼC whereas it showed highest growth at 25ᴼC as compared to 15ᴼC. Notably, it exhibited a wide range of pH from acidic to alkaline conditions (pH 4 to 10), with the most favorable pH for optimal growth being pH 6.0. Additionally, the strain revealed its endurance in NaCl concentrations of up to 2.0% (Supplementary Figure S1). To investigate the growth characteristics and cold tolerance of *Glaciimonas* sp. PAMC28666, a growth curve analysis was conducted at different temperatures. The species did not exhibit growth at 37°C. However, at 25°C, it reached the stationary phase after 72 h of incubation, and at 15°C, it reached the stationary phase after 96 h (Figure 1). Interestingly, it was found that there were no significant differences in the final cell density at the stationary phase between the two temperatures (data not shown). These results suggest that strain PAMC28666 might be categorized as a psychrotolerant bacterium.

### Genomic features of *Glaciimonas* sp. PAMC28666

The Antarctica isolate *Glaciimonas* sp. PAMC28666 is the only complete genome that has been deposited in the NCBI database until now. It comprises a single contig, with a total size of 5.2 Mb with 4,476 genes, a G + C content of 51.5% and genome coverage of 100.0X with reference sequences. Among these, 4,350 are protein-coding genes, while 12 are involved in ribosomal RNA (rRNA), along with 56 genes responsible for transfer RNA (tRNA), and 54 pseudogenes. It is noteworthy that the pseudogenes account for less than 2% of the entire gene set (See Supplementary Table S1). In the study conducted by Zhang et al. (2011) on *Glaciimonas immobilis*, a similar trait was noted within the genus *Glaciimonas* sp. PAMC28666*. Glaciimonas* sp. PAMC28666 showed a G + C content of 51.1% similar to *Glaciimonas immobilis* (Zhang et al., 2011) (Table 1). Additionally, as reported by Frasson et al. (2015) and Margesin et al. (2016), strain PAMC28666 shows an intermediate G + C content when compared to two *Glaciimonas* species, *Glaciimonas alpina* (49.2%) and *Glaciimonas frigoris* (53%). However, it is important to note that the G + C content within the genus *Collimonas* exhibits notable deviation, ranging from 56.06 to 59.6% (Song et al., 2015), significantly higher than that observed in the genus *Glaciimonas*.

**Table 1:** Comparison of genomic features (ANI, DDH, G + C content, and 16 s rRNA) of *Glaciimonas* sp. PAMC28666 with the closest members.

Parameters	Microorganisms							
	*G.* sp. PAMC28666^*^	*G. imobilis^T^*	*G. frigoris^T^*	*G. alpina^T^*	*G.* sp. PCH181	*C. fungivorans*Ter6^T^	*C. pratensis* Ter91^T^	*C. arenae* Ter10^T^
Isolation source	Soil of Antarctica	Alpine glaciers	Permafrost sediment	Alpine glaciers	Glacier water of India	Dune grassland	Dune grassland	Dune grassland
RefSeq	GCF016917355.1	–	–	–	GCF003056055.1	GCF001584145.1	GCF001584185.1	GCF001584165.1
GS (Mb)	5.23	–	–	–	5.3	5.6	5.7	4.7
ANI (%)	100	–	–	–	77.08	74	73.91	73.91
DDH (%)	100	–	–	–	23.3	21.4	22	21.6
G + C (%)	51.5	51	53	49.2	51	59	58.7	56.38
16 s (%)	100	98.97	99.34	99.93	–	96.15	96.21	96.21

G., Glacimmonas; C., Collimonas; −, data not available; RefSeq, reference genome sequence; GS, genome size; and *, the studied strain. Information: G + C content for G. frigoris, G. alpina and G, singularis were taken from the literature and for Glaciimonas sp. PCH181, and Collimonas G + C data were obtained from NCBI.

### Phylogenetic tree analysis of *Glaciimonas* sp. PAMC28666

**Figure 2:**
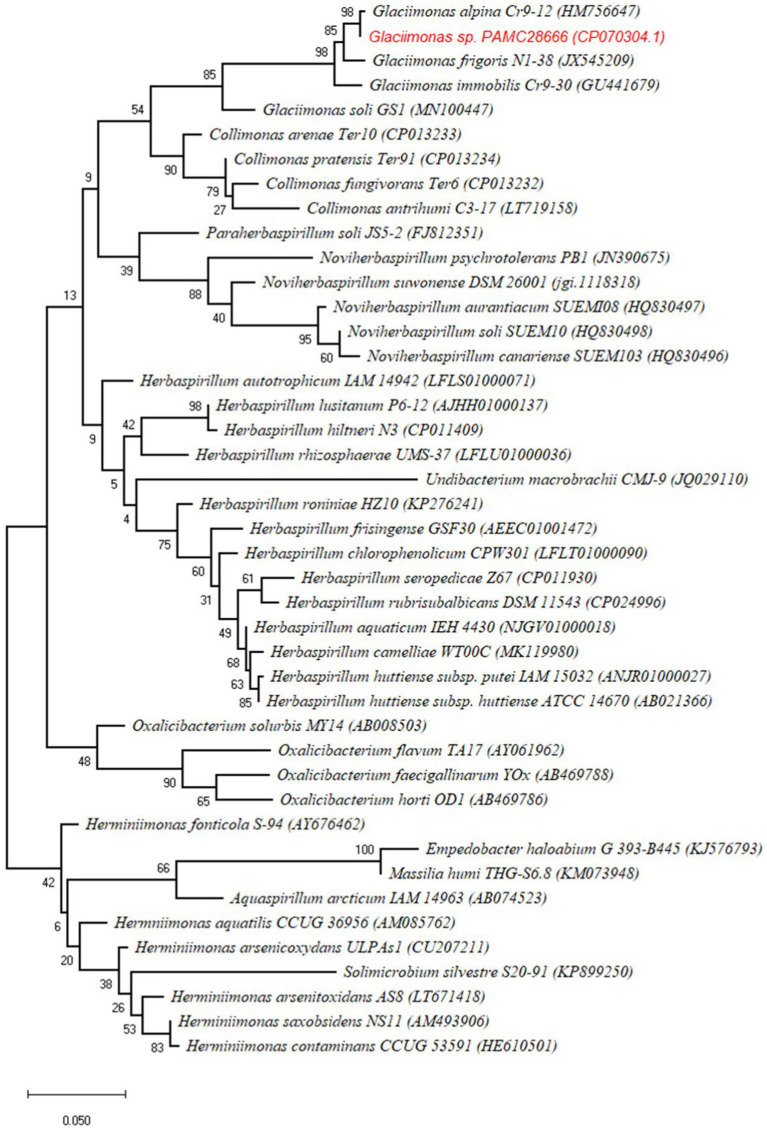
Phylogenetic tree of *Glaciimonas* sp. PAMC28666 with related strains based on 16 s rRNA sequences. The tree was constructed by maximum likelihood using MEGA X with 1,000 bootstrapping replications.

When analyzing the 16S rRNA gene sequence of *Glaciimonas* sp. PAMC28666 isolate, its closest matches were *Glaciimonas aplina* and *Glaciimonas frigoris*, sharing a remarkably high similarity of 99%. The subsequent closest match was identified as *Glaciimonas immobilis,* showing a similarity of 98.97%. However, it is crucial to emphasize the 16S rRNA sequence-based similarity of *Glaciimonas* sp. PAMC28666 exceeds the defined threshold value of 98.65% which is recommended for species differentiation (Kim et al., 2014). Furthermore, during phylogenetic analysis, it became evident that strain PAMC28666 occupies a unique branch within the genus *Glaciimonas,* displaying a notable proximity to *Glaciimonas* alpine Cr9-12 in the same clade. The presence of robust bootstrap values strengthens the validity of this clustering and reinforces its close affiliation with the genus *Glaciimonas* (Figure 2). Due to the unavailable of complete genome for the genus *Glaciimonas* except our strain PAMC28666, additional computational analysis was carried out involving digital DNA–DNA hybridization (DDH) and average nucleotide identity (ANI), focusing on members of the genus *Collimonas*, specifically *Collimonas fungivorans* strain Ter6^T^, *Collimonas pratensis* strain Ter91^T^, and *Collimonas arenae* strain Ter10^T^. The DDH values were found to vary between 21.4 and 22%, whereas the ANI values ranged from 73.91 to 74% (Table 1). In addition, strain PAMC28666 showed 77.08 and 23.3% ANI and DDH, respectively, with *Glaciimonas* sp. PCH181 (Kumar et al., 2020). These results suggest that strain PAMC28666 belongs to the genus to *Glaciimonas* rather than the genus *Collimonas.*

### Genomic insights revealed stress adaptation strategies in *Glaciimonas* sp. PAMC28666

The stress adaptation mechanisms of *Glaciimonas* sp. PAMC28666 has been studied, revealing a range of proteins that could potentially be expressed in response to various stress conditions. These conditions include cold stress, which triggers the expression of proteins like cold-shock proteins (CSPs), as well as chaperonins (GroEL/GroES, HscA/HscB, and SurA), and cold-active chaperones. Additionally, for osmotic stress, proteins such as choline dehydrogenase, sarcosine oxidase, ABC transporters, and permease for glycine/betaine/choline were detected. Regarding oxidative stress, the strain possesses antioxidant genes like superoxide dismutase, catalase, alkyl hydro-peroxidase, glutathione-peroxidase, peroxiredoxin, and thioredoxin family proteins. Further details regarding stress-related proteins are outlined in Supplementary Table S2. A comprehensive examination of cold-shock proteins was conducted to better understand their role in the cold stress adaptation of strain PAMC28666. In comparison to the reference strain *Escherichia coli* K12, these proteins, which contribute to elevated protein synthesis at lower temperatures, displayed a similarity of more than 50% (Table 2). The key genes CSPs, such as *cspA*, *cspB*, *cspC*, *cspD*, and *cspE*, exhibited identities of 63.08, 64.25, 68.25, 68.75, and 70.49%, respectively, with strain PAMC28666. Besides the key genes of CSPs, trehalose biosynthetic genes like trehalose phosphate synthase (*otsA*) and trehalose phosphatase (*otsB*) were also identified. However, their similarities with the reference strain *Escherichia coli* K12 were comparatively lower, measuring 45.89 and 38.43%, respectively. The presence of these genes indicates the potential for trehalose production within strain PAMC28666.

**Table 2:** List of genes encoding for proteins induced under cold stress conditions in the genome of *Glaciimonas* sp. PAMC28666.

NCBI reference sequence	Description	% identity with *E. coli* K12	References
WP_205319425.1	Pyruvate dehydrogenase, decarboxylase (AceE)	59.15	Jones and Inouye (1994)
WP_205319426.1	Pyruvate dehydrogenase, dihydrolipoamide acetyltransferase (AceF)	54.11	Jones and Inouye (1994)
WP_205321136.1	Cold-inducible RNA chaperone, transcriptional enhancer CspA	63.08	Goldstein et al. (1990) and Gualerzi et al. (2003)
WP_168052175.1	Function unknown (CspB)	64.52	Etchegaray et al. (1996)
WP_205319579.1	Regulation of expression of stress response proteins RpoS and UspA (CspcC)	68.25	Phadtare and Inouye (2001) and Shenhar et al. (2012)
WP_168057001.1	Biofilm development, inhibition of DNA replication (CspD)	68.75	Yamanaka and Inouye (2001a) and Kim et al. (2010)
WP_168052175.1	Regulation of expression of stress response proteins RpoS and UspA (CspE)	70.49	Phadtare and Inouye (2001), Shenhar et al. (2012), and Czapski and Trun (2014)
WP_205320165.1	Degradation of RNA (PNP)	64.97	Yamanaka and Inouye (2001b)
WP_205320931.1	Trehalose phosphate synthase (otsA)	45.89	Kandror et al. (2002)
WP_205320933.1	Trehalose phosphatase (OtsB)	38.43	Kandror et al. (2002)
WP_014004414.1	Protein chain initiation factor IF1 (InfA)	75.00	Gualerzi et al. (2003)
WP_205319525.1	Protein chain initiation factor IF2, binding of charged tRNA-fmet to 30S ribosomal subunit (InfB)	62.93	Gualerzi et al. (2003)
WP_108439777.1	Protein chain initiation factor IF3, mRNA translation stimulation (InfC)	64.53	Gualerzi et al. (2003)
WP_205321223.1	Protein-folding chaperone, ribosome binding (Tig)	40.71	Kandror et al. (2002)

### *In silico* analysis of trehalose biosynthesis in the genome of *Glaciimonas* sp. PAMC28666

**Figure 3:**
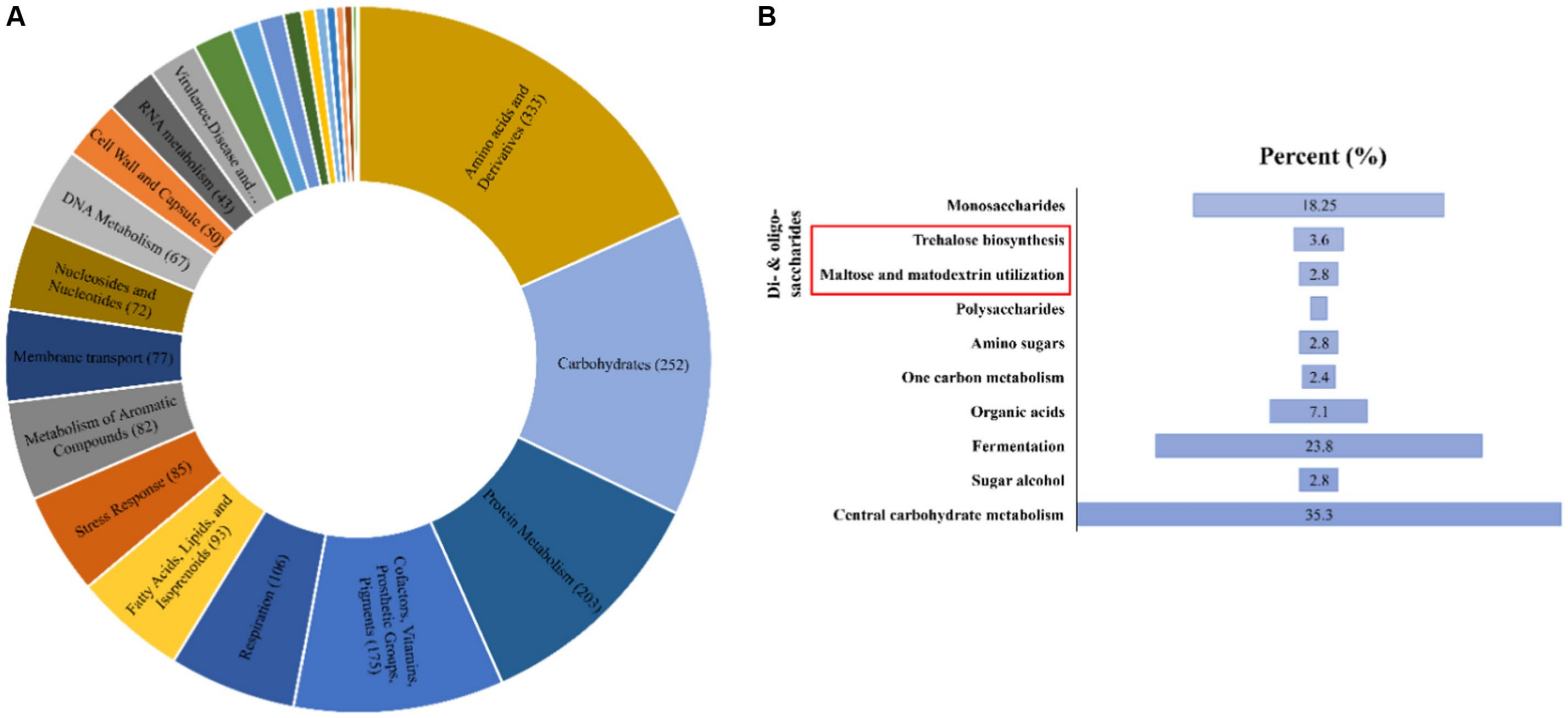
**(A)** A Pie chart illustrates the distribution of genes within different subsystem categories in the genome of *Glaciimonas* sp. PAMC28666; **(B)** A bar graph highlights the proportion of genes related to trehalose biosynthesis (indicated by a red box, constituting 3.6% of the total) within the Carbohydrate categories. The SEED viewer in the RAST server was used to gather data on the subsystem categories.

**Figure 4:**
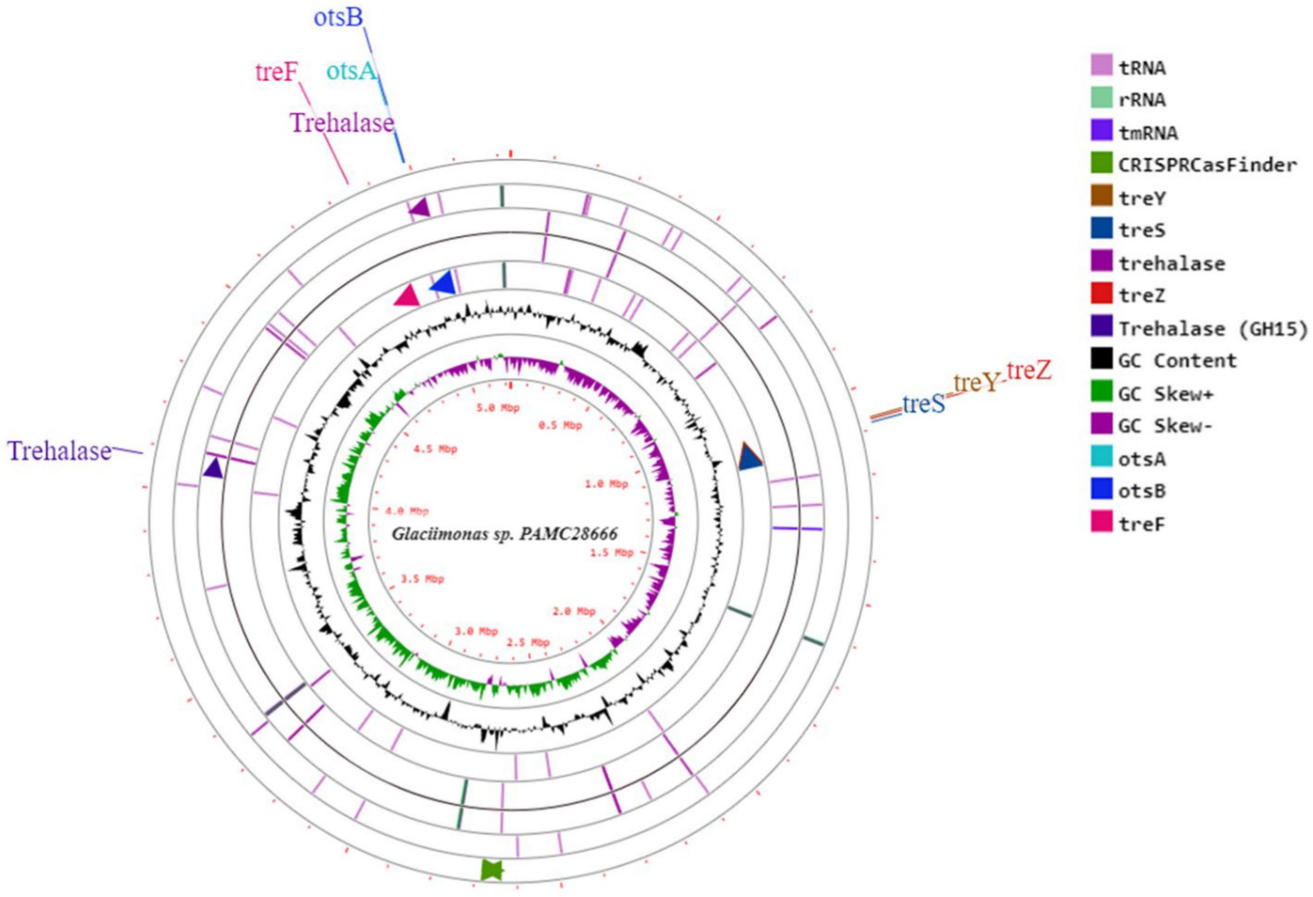
A circular depiction illustrates the genome and characteristics of *Glaciimonas* sp. PAMC28666. The components of the displayed rings (starting from the outermost to the center) are described as follows: Ring 1 contains CDS segments, encompassing tRNA and rRNA, along with Pokka annotation featuring genes linked to trehalose metabolism; Ring2 shows the combined ORFs in both forward and reverse strands; Ring 3 represents a plot indicating the GC content distribution; Ring 4 displays a GC skew plot, with values above average depicted in green and values below average depicted in purple; Ring 5 serves as a sequence ruler, offering a scale reference.

**Figure 5:**
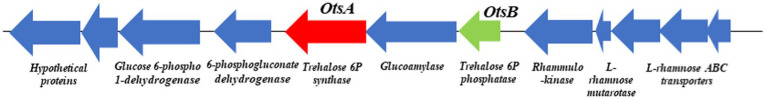
Genetic organizations of the *otsAB* gene clusters found in *Glaciimonas* sp. PAMC28666, i.e., *otsA* (encoding a trehalose-6-phosphate synthase) and *otsB* (encoding a trehalose-6-phosphate phosphatase). The arrows indicate the direction of gene transcription.

The Antarctica isolate *Glaciimonas* sp. PAMC28666 utilizes trehalose biosynthesis genes as a part of its response to cold stress through the analysis of the cold-shock protein was determined. To determine these genes involved in trehalose biosynthesis, several bioinformatics tools were employed. After conducting a RAST analysis, detected 325 subsystems. Among the prominent subsystems identified, the one with the most genes is the amino acid and derivatives system, totaling 333 genes. This is succeeded by carbohydrates with 252 genes, and respiration with 106 genes (Figure 3). Upon additional investigation, it was discovered that the studied strain possesses the di- and oligosaccharides metabolism under the carbohydrate subsystem, encompassing 3.6% of genes related to trehalose metabolism. Furthermore, the CGview web server is utilized to display the anticipated trehalose metabolism genes in the strain PAMC28666 (Figure 4). By using the KEGG annotation database, at first the pathways related to trehalose synthesis and their usefulness in this situation were evaluated. This study revealed that the trehalose biosynthetic pathways OtsA/B, TreYZ, and TS are present in the Antarctic isolate PAMC28666 (Supplementary Figure S2). It is significant that this mechanism also involves the OtsA/B pathway, which was previously identified as being essential to the cold shock protein’s function. Additionally, the presence of *otsA* and *otsB* genes were identified within the RAST annotation using the seed viewer, spanning a length of 1,413 and 795 coding sequences (CDS) respectively (Figure 5). Therefore, this validated the existence of the OtsA/B pathway for trehalose synthesis in the Antarctic strain PAMC28666.

### Freeze–thaw and cold shock effects on survival of *Glaciimonas* sp. PAMC28666

**Figure 6:**
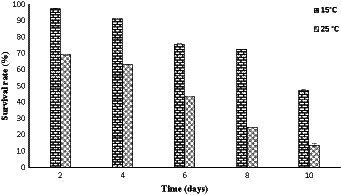
Effect of freeze–thaw times on the survival rates of *Glaciimonas* sp. PAMC28666.

A freeze–thaw cycle was conducted on strain PAMC28666 to evaluate its viability when exposed to cold conditions. In exploring the survival mechanism in a cold environment, it was observed that strain PAMC28666 exhibited greater viability at 15°C when compared to 25°C following the execution of freeze–thaw cycles. Furthermore, upon conducting three freeze–thaw cycles, it revealed that survival rates of 75.65% at 15°C and 43.29% at 25°C (Figure 6). However, following five freeze–thaw cycles, the survival rate decreased to 47.28% at 15°C, whereas at 25°C, it dropped significantly to only 13.69%. significantly, a noticeable reduction in the survival rate was witnessed following freeze-thaw cycles at 25°C, suggesting an increased susceptibility to cold stress at this specific condition.

### Determination the presence of trehalose

**Figure 7:**
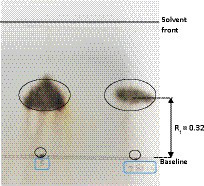
Analyses of the carbohydrates in the strain PAMC28666 cell extract. Note: To produce the cell extract, PAMC28666 cells cultured in R2A (100 mL, OD =2.0) were extracted with hot water. A TLC plate was spotted with ten microliters of the cell extract, which was then developed with n-butanol: ethanol: water = 5:3:2. Spraying 20% sulfuric acid in 5% ethanol was used to create the visualization. “Lane S” represents a standard sample of trehalose (Rf = 0.320), while “Lane 28666” corresponds to cell extracts from PAMC28666 (Rf = 0.32).

**Figure 8:**
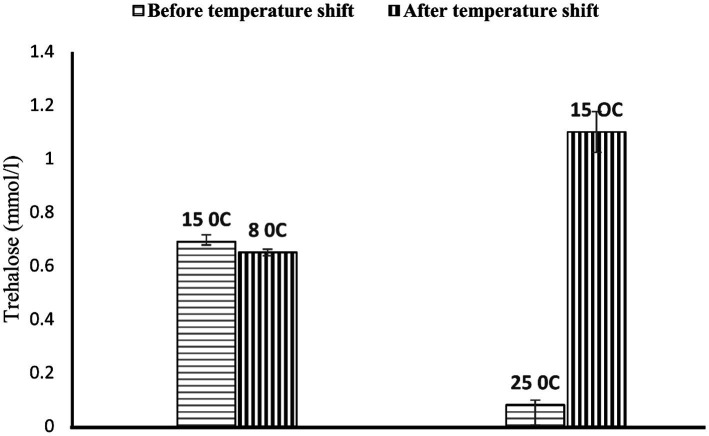
Accumulation of trehalose at 15 and 25°C correlates upon further temperature downshift to 8 and 15°C, respectively. The cells were grown to mid-logarithmic phase at each temperature 15 and 25°C, respectively, and then transferred to 8 and 15°C, respectively. At a steady growth phase at respective temperatures, aliquots were taken to measure intracellular trehalose content.

On further study, strain PAMC28666 showed the potential to synthesize trehalose at a lower temperature of 15°C. To confirm this finding, further conducted the thin layer chromatography (TLC) analysis which yielded a positive result with an Rf value correspondence with that of the standard trehalose (Figure 7). In addition, a commercial trehalase enzyme was utilized for verification. This observation indicated the possibility that strain PAMC28666 could be utilizing the OtsA/B pathway for trehalose production. Additionally, under the investigation of trehalose accumulation in strain PAMC28666 as a physiological response to a temperature decrease, it was noticed variations in the trehalose levels throughout the acclimation phase. Interestingly, as shifted the strain PAMC28666 cells from higher temperatures to lower ones, it showed two distinct outcomes. Initially, when transitioning from one psychrophilic condition (15°C) to another (8°C), strain PAMC28666 exhibited a negligible decrease in trehalose accumulation. Conversely, when transitioning from a mesophilic state (25°C) to psychrophilic conditions (15°C), we witnessed a remarkable approximately 13-fold rise in trehalose accumulation (Figure 8). These results indicate that Antarctic isolate *Glaciimonas* sp. PAMC28666 could synthesize trehalose under temperature-induced stress conditions, thereby providing further confirmation of the involvement of the OtsA/B pathway.

## Discussion

The strain PAMC28666 from Antarctica was obtained from a soil sample from the Antarctica region. It comes from an environment with temperatures lower than 10°C. The identification of the strain PAMC28666 as a member of the genus *Glaciimonas* was based on comparing its 16S rRNA sequence with known sequences. The phylogenetic analysis further supported the relatedness of PAMC28666 to the genus *Glaciimonas*. According to the results of species classification using average nucleotide (ANI) and DNA–DNA hybridization (DDH) values, PAMC28666 did not meet the threshold for species classification (Richter and Rosselló-Móra, 2009). Moreover, the G + C content and 16S rRNA sequence similarity of PAMC28666 aligned with the phylogenetic groupings of strains *Glaciimonas*. The genus *Glaciimonas* is situated in the *Oxalobacteraceae* family within the Betaproteobacteria class. It encompasses four well-defined species: *Glaciimonas immobilis, Glaciimonas alpina, Glaciimonas frigoris*, and *Glaciimonas singularis*. These species have been isolated from diverse glacial environments globally (Zhang et al., 2011; Frasson et al., 2015; Margesin et al., 2016), and in the case of *Glaciimonas singularis*, from a water sample in a uranium mine (Chung et al., 2013). This information indicates that the *Glaciimonas* sp. PAMC28666 strain is a novel cold-adapted bacterium indigenous to the Antarctic region. Notably, it is the only complete genome of genus *Glaciimonas* deposited in the NCBI database until now. Although this strain was originally isolated from an extremely cold region, it has shown the ability to thrive in a culture medium at both 15°C and 25°C (Supplementary Figure S1). This adaptability to grow under such diverse temperature conditions suggest its resilience to both cold and warm environments, classifying it as a psychrotroph. Moreover, strain PAMC28666 demonstrated resistance to salt which exhibited the potential to adapt to osmotic stress.

A noteworthy discovery related to the cold-resistant bacteria pertains to the existence of specific proteins that facilitate adaptation to cold conditions. Similar cold stress proteins have been identified within the Antarctic strain PAMC28666 (Table 2). In response to these challenges, bacteria induce the synthesis of diverse proteins, which encompass cold shock proteins, cold-active chaperones, and chaperonins. CSPs, characterized by their small size, engage with nucleic acids and are present across various microorganisms, including psychrophiles (organisms thriving in cold environments), mesophiles (organisms thriving in moderate temperatures), and thermophiles (organisms thriving in high temperatures) (Czapski and Trun, 2014; Jin et al., 2014). Different bacteria may exhibit varying types and levels of CSP expression in response to cold shock. Among the CSP genes, *cspA* is highly expressed and serves as an essential mRNA chaperone, regulating the transcription of other cold shock genes (Keto-Timonen et al., 2016) (Figure 6).

Moreover, there have been reports indicating that certain bacteria possess multiple paralogs (related genes) of CSPs within their genomes, and these paralogs can be regulated independently. Within this context, distinct paralogs might be induced by low temperatures, during the stationary growth phase, or could exhibit constitutive expression (Yamanaka et al., 1994; Yamanaka and Inouye, 1997; Graumann and Marahiel, 1998; Yamanaka et al., 1998; Graumann and Marahiel, 1999; Wang et al., 1999). In strain PAMC28666, significant genes associated with cold shock proteins, *including cspA, cspB, cspC, cspD,* and *cspE* were identified. Additionally, genes related to trehalose biosynthesis, such as trehalose phosphate synthase (*otsA*) and trehalose phosphatase (*otsB*) were also detected. This suggests that strain PAMC28666 possesses the capability to synthesize trehalose under conditions of cold stress. Additionally, cold acclimation proteins, often referred to as “CAPs,” play a pivotal role in augmenting protein synthesis within bacteria under conditions of low temperatures (Margesin, 2007). These proteins are commonly present in bacteria inhabiting consistently cold environments and serve as markers for identifying psychrophiles (Mishra et al., 2010; Piette et al., 2011). As a result, it was hypothesized that strain PAMC28666 might have potentially to endure such extreme environments due to the presence of these CAPs. Furthermore, on further analysis into the mechanisms behind bacterial survival during cold stress, there have been prior investigations into the presence of compatible solutes within certain bacteria. Compatible solutes encompass a group of small molecules that can accumulate within cells without disrupting their essential biological functions. These solutes have been identified as cold-protective agents, including trehalose, glycine betaine, and carnitine. They are known to accumulate in response to cold shock, either through increased synthesis or uptake. In *Escherichia coli*, the genes *otsA* and *otsB* are responsible for the biosynthesis of trehalose from glucose, encoding trehalose 6-phosphate synthase and trehalose-6-phosphate phosphatase, respectively (Giaever et al., 1988). The OtsAB operon can be induced during cold shock, osmotic stress, and the stationary phase, and its expression is regulated by the Rpos protein (Giaever et al., 1988; Hengge-Aronis et al., 1991; White-Ziegler et al., 2008). In the case of *Glaciimonas* sp. PAMC28666, it was observed that trehalose also accumulates during cold shock, specifically at 15°C. While trehalose content did not impact growth at 25°C or 15°C, higher levels of trehalose enhanced viability under freezing and thawing conditions. Conversely, a lack of trehalose correlated with a reduced ability to survive under these conditions. Trehalose accumulation during the cold-shock response is thought to be part of the cell’s adaptation to the temperature drop, and its accumulation follows a similar time course to known cold-shock proteins (Jones et al., 1987). Trehalose, a vital compound for organisms in stressful environments due to its distinct physiochemical properties, serves to safeguard cellular integrity against various environmental harms and nutritional limitations (Luyckx and Baudouin, 2011). In addition, it has been also reported that trehalose biosynthetic pathways appear to be species-specific (Tournu et al., 2013).

It has been also reported that apart from transcriptional changes, the stability of mRNA molecules is an important factor in the induction of cold-shock mRNA expression at low temperatures (Fang et al., 1997). For example, the half-life of *otsAB* mRNA, responsible for trehalose synthesis, increases from less than 2 min at 37°C to approximately 20 min at 16°C (Kandror et al., 2002). This increase in *otsA/B* mRNA levels, occurring before trehalose accumulation, likely contributes to its buildup during cold shock and suggests enhanced transcription of the *otsA/B* genes. This might correlate to the findings as strain PAMC28666 was able to produce more trehalose when transferred from high temperature to low temperature.

**Figure 9:**
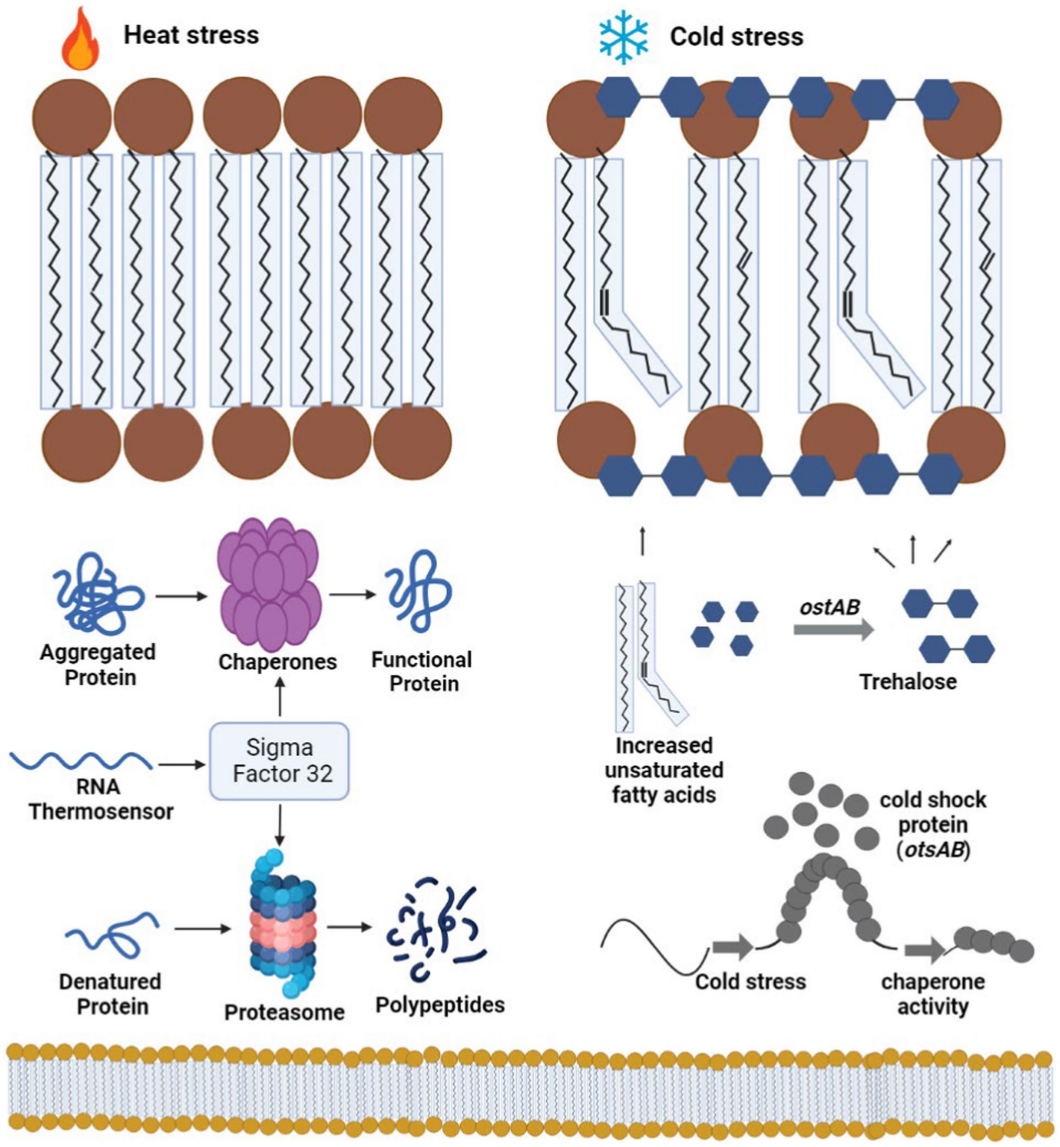
A schematic illustrates a simplified mode of action of cold shock proteins. When challenged with low-temperature stress, bacteria initiate a dramatic accumulation of cold shock proteins. Bacteria cold shock proteins function to effectively melt double-stranded regions of RNA transcripts. Cold shock genes, such *otsAB*, produce trehalose in the presence of glucose molecules, which acts as an intercalating agent between the membrane, protecting it from cold bursts. The figure was created with BioRender.com.

Trehalose has been extensively studied for its capacity to improve cell viability under diverse temperature conditions. At high temperatures, it acts as a “chemical chaperone,” protecting cells by mitigating protein denaturation and aggregation induced by heat stress (De Virgilio et al., 1994; Welch and Brown, 1996; Singer and Lindquist, 1998). Moreover, trehalose may have a role in stabilizing cell membranes at 4°C, as lower temperatures lead to reduced fluidity (Figure 9). Interestingly, exogenous trehalose has been shown to safeguard various organisms from freezing (Lodato et al., 1999; Eroglu et al., 2000), and its maximum protective effect is observed when it is present on both sides of the cell membrane (Diniz-Mendes et al., 1999; Eroglu et al., 2000). The mechanisms underlying trehalose’s ability to enhance cell viability at low temperatures likely involve multiple processes, potentially distinct from those proposed previously.

## Conclusion

*Glaciimonas* sp. PAMC28666 is a unique aerobic bacterium that has been found in Antarctica and exhibits psychrotolerant characteristics. In this study, we present the complete genome of genus *Glaciimonas* that is deposited in the NCBI database as *Glaciimonas* sp. PAMC28666. The findings show that this bacterium could adapt to freezing temperatures while retaining its aerobic characteristics. Through complete genome analysis, a significant understanding of the complex physiological and metabolic functions of *Glaciimonas* sp. PAMC28666 has been attained. The finding of genes linked to both oxidative and cold stress is particularly intriguing since it suggests that the bacterium can adapt to stressful environments. Furthermore, it was found that gene clusters are responsible for the biosynthesis of trehalose via the *otsAB* genes. Experimental validation has effectively confirmed the bacterium’s capacity to adjust to cold conditions. Additionally, our investigation revealed the pivotal role of temperature as a determining factor that likely influences the regulation of key genes within bacterial systems. It is essential to highlight that this complete genome, the only one present in the NCBI repository, offers comprehensive insights into *Glaciimonas* genome. This information is pivotal for further deciphering the mechanisms behind its survival in extreme environments. Moreover, this knowledge could potentially pave the way for subsequent investigations into the importance of cold-adapted proteins in various biotechnological applications.

## Data availability statement

The datasets presented in this study can be found in online repositories. The names of the repository/repositories and accession number(s) can be found in the article/Supplementary material.

## Author contributions

PS: Conceptualization, Formal analysis, Investigation, Writing – original draft, Writing – review & editing. JK: Conceptualization, Formal analysis, Investigation, Writing – original draft, Writing – review & editing. S-RH: Investigation, Writing – original draft, Writing – review & editing. JL: Writing – original draft, Writing – review & editing. T-JO: Conceptualization, Funding acquisition, Project administration, Resources, Supervision, Writing – original draft, Writing – review & editing.

## Footnotes

^1^ http://github.com/pacificbiosciences/SMRT-Analysis

^2^ http://www.ezbiocloud.net/

^3^ http://ggdc.dsmz.de/

## References

[ref1] Aziz R. K. Bartels D. Best A. DeJongh M. Disz T. Edwards R. A.. (2008). The RAST server: rapid annotations using subsystems technology. BMC Genomics 9:75. doi: 10.1186/1471-2164-9-75, PMID: 18261238 PMC2265698

[ref2] Berger F. Morellet N. Menu F. Potier P. (1996). Cold shock and cold acclimation proteins in the psychrotrophic bacterium *Arthrobacter globiformis* SI55. J. Bacteriol. 178, 2999–3007. doi: 10.1128/jb.178.11.2999-3007.1996, PMID: 8655472 PMC178044

[ref3] Bylund G. O. Wipemo L. C. Lundberg L. A. C. Wikström P. M. (1998). RimM and RbfA are essential for efficient processing of 16S rRNA in *Escherichia coli*. J. Bacteriol. 180, 73–82. doi: 10.1128/jb.180.1.73-82.1998, PMID: 9422595 PMC106851

[ref4] Chin C. S. Alexander D. H. Marks P. Klammer A. A. Drake J. Heiner C.. (2013). Nonhybrid, finished microbial genome assemblies from long-read SMRT sequencing data. Nat. Methods 10, 563–569. doi: 10.1038/nmeth.2474, PMID: 23644548

[ref5] Chung A. P. Tiago I. Nobre M. F. Veríssimo A. Morais P. V. (2013). *Glaciimonas singularis* sp. nov., isolated from a uranium mine wastewater treatment plant. Int. J. Syst. Evol. Microbiol. 63, 2344–2350. doi: 10.1099/ijs.0.046581-0, PMID: 23178726

[ref6] Collins T. Margesin R. (2019). Psychrophilic lifestyles: mechanisms of adaptation and biotechnological tools. Appl. Microbiol. Biotechnol. 103, 2857–2871. doi: 10.1007/s00253-019-09659-5 30729286

[ref7] Czapski T. R. Trun N. (2014). Expression of *csp* genes in *E. coli* K-12 in defined rich and defined minimal media during normal growth, and after cold-shock. Gene 547, 91–97. doi: 10.1016/j.gene.2014.06.033, PMID: 24952137

[ref8] De Virgilio C. Hottiger T. Dominguez J. Boller T. Wiemken A. (1994). The role of trehalose synthesis for the acquisition of thermotolerance in yeast: I. Genetic evidence that trehalose is a thermoprotectant. Eur. J. Biochem. 219, 179–186. doi: 10.1111/j.1432-1033.1994.tb19928.x 8306984

[ref9] Diniz-Mendes L. Bernardes E. De Araujo P. S. Panek A. D. Paschoalin V. M. F. (1999). Preservation of frozen yeast cells by trehalose. Biotechnol. Bioeng. 65, 572–578. doi: 10.1002/(SICI)1097-0290(19991205)65:5<572::AID-BIT10>3.0.CO;2-7, PMID: 10516583

[ref10] Eroglu A. Russo M. J. Bieganski R. Fowler A. Cheley S. Bayley H.. (2000). Intracellular trehalose improves the survival of cryopreserved mammalian cells. Nat. Biotechnol. 18, 163–167. doi: 10.1038/72608 10657121

[ref11] Etchegaray J. P. Jones P. G. Inouye M. (1996). Differential thermoregulation of two highly homologous cold-shock genes, *cspA* and *cspB*, of *Escherichia coli*. Genes Cells 1, 171–178. doi: 10.1046/j.1365-2443.1996.d01-231.x, PMID: 9140061

[ref12] Fang L. Jiang W. Bae W. Inouye M. (1997). Promoter-independent cold-shock induction of *cspA* and its derepression at 37°C by mRNA stabilization. Mol. Microbiol. 23, 355–364. doi: 10.1046/j.1365-2958.1997.2351592.x 9044269

[ref13] Frasson D. Udovičić M. Frey B. Lapanje A. Zhang D. C. Margesin R.. (2015). *Glaciimonas alpina* sp. nov. isolated from alpine glaciers and reclassification of *Glaciimonas immobilis* Cr9-12 as the type strain of *Glaciimonas alpina* sp. nov. Int. J. Syst. Evol. Microbiol. 65, 1779–1785. doi: 10.1099/ijs.0.000174, PMID: 26184665

[ref14] Giaever H. M. Styrvold O. B. Kaasen I. Strøm A. R. (1988). Biochemical and genetic characterization of osmoregulatory trehalose synthesis in *Escherichia coli*. J. Bacteriol. 170, 2841–2849. doi: 10.1128/jb.170.6.2841-2849.1988, PMID: 3131312 PMC211211

[ref15] Goldstein J. Pollitt N. S. Inouye M. (1990). Major cold shock protein of *Escherichia coli*. Proc. Natl. Acad. Sci. U. S. A. 87, 283–287. doi: 10.1073/pnas.87.1.283, PMID: 2404279 PMC53247

[ref16] Grant J. R. Stothard P. (2008). The CGView server: a comparative genomics tool for circular genomes. Nucleic Acids Res. 36, W181–W184. doi: 10.1093/nar/gkn179, PMID: 18411202 PMC2447734

[ref17] Graumann P. L. Marahiel M. A. (1998). A superfamily of proteins that contain the cold-shock domain. Trends Biochem. Sci. 23, 286–290. doi: 10.1016/S0968-0004(98)01255-9, PMID: 9757828

[ref18] Graumann P. L. Marahiel M. A. (1999). Cold shock proteins CspB and CspC are major stationary-phase-induced proteins in *Bacillus subtilis*. Arch. Microbiol. 171, 135–138. doi: 10.1007/s002030050690, PMID: 9914312

[ref20] Gualerzi C. O. Giuliodori A. M. Pon C. L. (2003). Transcriptional and post-transcriptional control of cold-shock genes. J. Mol. Biol. 331, 527–539. doi: 10.1016/S0022-2836(03)00732-0, PMID: 12899826

[ref21] Hébraud M. Potier P. (1999). Cold shock response and low temperature adaptation in psychrotrophic bacteria. J. Mol. Microbiol. Biotechnol. 1, 211–219. PMID: 10943552

[ref22] Hengge-Aronis R. Klein W. Lange R. Rimmele M. Boos W. (1991). Trehalose synthesis genes are controlled by the putative sigma factor encoded by *rpoS* and are involved in stationary-phase thermotolerance in *Escherichia coli*. J. Bacteriol. 173, 7918–7924. doi: 10.1128/jb.173.24.7918-7924.1991, PMID: 1744047 PMC212585

[ref23] Jin B. Jeong K. W. Kim Y. (2014). Structure and flexibility of the thermophilic cold-shock protein of *Thermus aquaticus*. Biochem. Biophys. Res. Commun. 451, 402–407. doi: 10.1016/j.bbrc.2014.07.127, PMID: 25101648

[ref24] Jones P. G. Inouye M. (1994). The cold-shock response - a hot topic. Mol. Microbiol. 11, 811–818. doi: 10.1111/j.1365-2958.1994.tb00359.x 8022259

[ref25] Jones P. G. VanBogelen R. A. Neidhardt F. C. (1987). Induction of proteins in response to low temperature in *Escherichia coli*. J. Bacteriol. 169, 2092–2095. doi: 10.1128/jb.169.5.2092-2095.1987, PMID: 3553157 PMC212099

[ref26] Kandror O. DeLeon A. Goldberg A. L. (2002). Trehalose synthesis is induced upon exposure of *Escherichia coli* to cold and is essential for viability at low temperatures. Proc. Natl. Acad. Sci. U. S. A. 99, 9727–9732. doi: 10.1073/pnas.142314099, PMID: 12105274 PMC124994

[ref27] Kanehisa M. Goto S. (2000). KEGG: Kyoto encyclopedia of genes and genomes. Nucleic Acids Res. 28, 27–30. doi: 10.1093/nar/28.1.27 10592173 PMC102409

[ref28] Keto-Timonen R. Hietala N. Palonen E. Hakakorpi A. Lindström M. Korkeala H. (2016). Cold shock proteins: a minireview with special emphasis on Csp-family of enteropathogenic yersinia. Front. Microbiol. 7:1151. doi: 10.3389/fmicb.2016.01151, PMID: 27499753 PMC4956666

[ref29] Kim M. Oh H. S. Park S. C. Chun J. (2014). Towards a taxonomic coherence between average nucleotide identity and 16S rRNA gene sequence similarity for species demarcation of prokaryotes. Int. J. Syst. Evol. Microbiol. 64, 346–351. doi: 10.1099/ijs.0.059774-0 24505072

[ref30] Kim Y. Wang X. Zhang X. S. Grigoriu S. Page R. Peti W.. (2010). *Escherichia coli* toxin/antitoxin pair MqsR/MqsA regulate toxin CspD. Environ. Microbiol. 12, 1105–1121. doi: 10.1111/j.1462-2920.2009.02147.x, PMID: 20105222 PMC3980499

[ref32] Klein W. Weber M. H. W. Marahiel M. A. (1999). Cold shock response of *Bacillus subtilis*: isoleucine-dependent switch in the fatty acid branching pattern for membrane adaptation to low temperatures. J. Bacteriol. 181, 5341–5349. doi: 10.1128/jb.181.17.5341-5349.1999 10464205 PMC94040

[ref33] Kumar S. Stecher G. Li M. Knyaz C. Tamura K. (2018). MEGA X: molecular evolutionary genetics analysis across computing platforms. Mol. Biol. Evol. 35, 1547–1549. doi: 10.1093/molbev/msy096, PMID: 29722887 PMC5967553

[ref34] Kumar V. Thakur V. Ambika Kumar V. Kumar R. Singh D. (2020). Genomic insights revealed physiological diversity and industrial potential for *Glaciimonas* sp. PCH181 isolated from *Satrundi glacier* in Pangi-Chamba Himalaya. Genomics 112, 637–646. doi: 10.1016/j.ygeno.2019.04.016, PMID: 31022438

[ref35] Larkin M. A. Blackshields G. Brown N. P. Chenna R. Mcgettigan P. A. McWilliam H.. (2007). Clustal W and Clustal X version 2.0. Bioinformatics 23, 2947–2948. doi: 10.1093/bioinformatics/btm404, PMID: 17846036

[ref36] Lodato P. Se Govia De Huergo M. Buera M. P. (1999). Viability and thermal stability of a strain of *Saccharomyces cerevisiae* freeze-dried in different sugar and polymer matrices. Appl. Microbiol. Biotechnol. 52, 215–220. doi: 10.1007/s002530051511, PMID: 10499261

[ref37] Luyckx J. Baudouin C. (2011). Trehalose: an intriguing disaccharide with potential for medical application in ophthalmology. Clin. Ophthalmol. 5, 577–581. doi: 10.2147/OPTH.S18827, PMID: 21654884 PMC3102588

[ref38] Margesin R. (2007). Alpine microorganisms: useful tools for low-temperature bioremediation. J. Microbiol. 45, 281–285. PMID: 17846579

[ref39] Margesin R. Zhang D. C. Frasson D. Brouchkov A. (2016). *Glaciimonas frigoris* sp. nov., a psychrophilic bacterium isolated from ancient Siberian permafrost sediment, and emended description of the genus *Glaciimonas*. Int. J. Syst. Evol. Microbiol. 66, 744–748. doi: 10.1099/ijsem.0.000783, PMID: 26597157

[ref40] Mei Y. Z. Huang P. W. Liu Y. He W. Fang W. W. (2016). Cold stress promoting a psychrotolerant bacterium *Pseudomonas fragi* P121 producing trehaloase. World J. Microbiol. Biotechnol. 32:134. doi: 10.1007/s11274-016-2097-1, PMID: 27339315

[ref41] Meier-Kolthoff J. P. Auch A. F. Klenk H. P. Göker M. (2013). Genome sequence-based species delimitation with confidence intervals and improved distance functions. BMC Bioinformatics 14:60. doi: 10.1186/1471-2105-14-60, PMID: 23432962 PMC3665452

[ref42] Miller G. L. Blum R. Glennon W. E. Burton A. L. (1960). Measurement of carboxymethylcellulase activity. Anal. Biochem. 1, 127–132. doi: 10.1016/0003-2697(60)90004-X

[ref43] Mishra P. K. Joshi P. Bisht S. C. Bisht J. K. Selvakumar G. (2010). “Cold-Tolerant Agriculturally Important Microorganisms,” in Plant Growth and Health Promoting Bacteria. Microbiology Monographs, Ed. Meheshwori D. (Berlin, Heidelberg: Springer), 18. doi: 10.1007/978-3-642-13612-2_12

[ref44] Pearce D. A. (2012). “Extremophiles in Antarctica: life at low temperatures” in Adaption of microbial life to environmental extremes, novel research results and application, 87–118.

[ref45] Phadtare S. Inouye M. (2001). Role of CspC and CspE in regulation of expression of RpoS and UspA, the stress response proteins in *Escherichia coli*. J. Bacteriol. 183, 1205–1214. doi: 10.1128/JB.183.4.1205-1214.2001, PMID: 11157932 PMC94993

[ref46] Piette F. D’Amico S. Mazzucchelli G. Danchin A. Leprince P. Feller G. (2011). Life in the cold: a proteomic study of cold-repressed proteins in the antarctic bacterium *Pseudoalteromonas haloplanktis* TAC125. Appl. Environ. Microbiol. 77, 3881–3883. doi: 10.1128/AEM.02757-10, PMID: 21478318 PMC3127592

[ref47] Richter M. Rosselló-Móra R. (2009). Shifting the genomic gold standard for the prokaryotic species definition. Proc. Natl. Acad. Sci. U. S. A. 106, 19126–19131. doi: 10.1073/pnas.0906412106, PMID: 19855009 PMC2776425

[ref48] Roberts M. E. Inniss W. E. (1992). The synthesis of cold shock proteins and cold acclimation proteins in the psychrophilic bacterium *Aquaspirillum arcticum*. Curr. Microbiol. 25, 275–278. doi: 10.1007/BF01575861

[ref49] Rogers J. S. Swofford D. L. (1998). A fast method for approximating maximum likelihoods of phylogenetic trees from nucleotide sequences. Syst. Biol. 47, 77–89. doi: 10.1080/106351598261049, PMID: 12064242

[ref50] Shenhar Y. Biran D. Ron E. Z. (2012). Resistance to environmental stress requires the RNA chaperones CspC and CspE. Environ. Microbiol. Rep. 4, 532–539. doi: 10.1111/j.1758-2229.2012.00358.x, PMID: 23760898

[ref51] Singer M. A. Lindquist S. (1998). Multiple effects of trehalose on protein folding in vitro and in vivo. Mol. Cell 1, 639–648. doi: 10.1016/S1097-2765(00)80064-7, PMID: 9660948

[ref52] Song C. Schmidt R. de Jager V. Krzyzanowska D. Jongedijk E. Cankar K.. (2015). Exploring the genomic traits of fungus-feeding bacterial genus Collimonas. BMC Genomics 16:1103. doi: 10.1186/s12864-015-2289-3, PMID: 26704531 PMC4690342

[ref53] Tournu H. Fiori A. Van Dijck P. (2013). Relevance of trehalose in pathogenicity: some general rules, yet many exceptions. PLoS Pathog. 9:e1003447. doi: 10.1371/journal.ppat.1003447, PMID: 23966851 PMC3744402

[ref54] Wang N. Yamanaka K. Inouye M. (1999). CspI, the ninth member of the CspA family of *Escherichia coli*, is induced upon cold shock. J. Bacteriol. 181, 1603–1609. doi: 10.1128/jb.181.5.1603-1609.1999, PMID: 10049393 PMC93551

[ref55] Welch W. J. Brown C. R. (1996). Influence of molecular and chemical chaperones on protein folding. Cell Stress Chaperones 1, 109–115. doi: 10.1379/1466-1268(1996)001<0109:IOMACC>2.3.CO;2 9222596 PMC248462

[ref56] White-Ziegler C. A. Um S. Pérez N. M. Berns A. L. Malhowski A. J. Young S. (2008). Low temperature (23 °C) increases expression of biofilm-, cold-shock- and RpoS-dependent genes in *Escherichia coli* K-12. Microbiology 154, 148–166. doi: 10.1099/mic.0.2007/012021-0, PMID: 18174134

[ref57] Yamanaka K. Fang L. Inouye M. (1998). The CspA family in *Escherichia coli*: multiple gene duplication for stress adaptation. Mol. Microbiol. 27, 247–255. doi: 10.1046/j.1365-2958.1998.00683.x, PMID: 9484881

[ref58] Yamanaka K. Inouye M. (1997). Growth-phase-dependent expression of *cspD*, encoding a member of the CspA family in *Escherichia coli*. J. Bacteriol. 179, 5126–5130. doi: 10.1128/jb.179.16.5126-5130.1997, PMID: 9260955 PMC179371

[ref59] Yamanaka K. Inouye M. (2001a). Induction of CspA, an *E. coli* major cold-shock protein, upon nutritional upshift at 37°C. Genes Cells 6, 279–290. doi: 10.1046/j.1365-2443.2001.00424.x 11318871

[ref60] Yamanaka K. Inouye M. (2001b). Selective mRNA degradation by polynucleotide phosphorylase in cold shock adaptation in *Escherichia coli*. J. Bacteriol. 183, 2808–2816. doi: 10.1128/JB.183.9.2808-2816.2001, PMID: 11292800 PMC99497

[ref61] Yamanaka K. Mitani T. Ogura T. Niki H. Hiraga S. (1994). Cloning, sequencing, and characterization of multicopy suppressors of a *mukB* mutation in *Escherichia coli*. Mol. Microbiol. 13, 301–312. doi: 10.1111/j.1365-2958.1994.tb00424.x, PMID: 7984109

[ref62] Yoon S. H. Ha S. M. Kwon S. Lim J. Kim Y. Seo H.. (2017). Introducing EzBioCloud: a taxonomically united database of 16S rRNA gene sequences and whole-genome assemblies. Int. J. Syst. Evol. Microbiol. 67, 1613–1617. doi: 10.1099/ijsem.0.001755, PMID: 28005526 PMC5563544

[ref63] Zhang D. C. Redzic M. Schinner F. Margesin R. (2011). *Glaciimonas immobilis* gen. Nov., sp. nov., a member of the family Oxalobacteraceae isolated from alpine glacier cryoconite. Int. J. Syst. Evol. Microbiol. 61, 2186–2190. doi: 10.1099/ijs.0.028001-0 20935085

