# Supplementary material for: Elucidation of cold adaptation in Glaciimonas sp. PAMC28666 with special focus on trehalose biosynthesis
Source: Front Microbiol. 2023 Oct 18;14:1280775. doi: 10.3389/fmicb.2023.1280775 (PMC10618363; doi:10.3389/fmicb.2023.1280775)
Supplement: Supplementary file 1 [file Data_Sheet_1.docx]

**Elucidation of cold adaptation in *Glaciimonas* sp. PAMC28666 with special focus on trehalose biosynthesis**

Prasansah Shrestha^1†^, Jayram Karmacharya^1†^, So-Ra Han^1,2,3^, Jun Hyuck Lee^4^, and Tae-Jin Oh^1,2,3,5,*^

^1^ Department of Life Sciences and Biochemical Engineering, Graduate School, SunMoon University, Asan 31460, South Korea; [prasansahshrestha@gmail.com](mailto:prasansahshrestha@gmail.com) (P. Shrestha) & [jayram.karmacharya@gmail.com](mailto:jayram.karmacharya@gmail.com) (J. Karmacharya)

^2^ Genome-based Bio-IT Convergence Institute, Asan 31460, South Korea; [553sora@hanmail.net](mailto:553sora@hanmail.net) (S.-R. Han)

^3^ Bio Big Data-based Chungnam Smart Clean Research Leader Training Program, SunMoon University, Asan 31460, South Korea

^4^ Research Unit of Cryogenic Novel Materials, Korea Polar Research Institute, Incheon 21990, South Korea; [junhyucklee@kopri.re.kr](mailto:junhyucklee@kopri.re.kr) (J.H. Lee)

^5^ Department of Pharmaceutical Engineering and Biotechnology, SunMoon University, Asan 31460, South Korea

^“†”^ These authors contributed equally to this work.

*Correspondence: tjoh3782@sunmoon.ac.kr (T.-J. Oh)

**Legends to supplementary tables**

**Table S1.** Genomic features of *Glaciimonas* sp. PAMC28666.

**Table S2.** List of genes encoding for proteins involved in cold and stress adaptation in the genome of *Glaciimonas* sp. PAMC28666.

**Table S1**

| **Attribute** | **Value** |
| --- | --- |
| Genome size | 5.23036 Mb |
| Coverage | 100 |
| G+C content | 51.5% |
| Contig | 1 |
| Total gene | 4476 |
| Protein coding gene | 4350 |
| rRNA gene | 4, 4, 4 (5S, 16S, 23S) |
| tRNA | 56 |
| Pseudo gene | 54 |
| Plasmid | 0 |

**Table S2**

| **NCBI reference sequence** | **Description** | **NCBI reference sequence** | **Description** |
| --- | --- | --- | --- |
| **Cold Stress**  WP_168057001.1,  WP_205321136.1 | Cold-shock proteins | **Osmotic Stress** WP_240162516.1, WP_240162580.1 | Glycine/betaine ABC transporter substrate-binding protein |
| WP_205321858.1 | ATP-dependent chaperone (ClpB) | WP_205323180.1 | Choline dehydrogenase |
| WP_205321858.1 | Molecular chaperone | WP_205319701.1 | Glycine betaine/L-proline ABC transporter ATP-binding protein |
| WP_205322086.1 | Co-chaperone (GroES) | WP_205321103.1, WP_205321132.1, WP_205321135.1 | ABC transporter ATP-binding protein |
| WP_205322087.1 | Chaperonin (GroEL) | WP_2053210991, WP_205323107.1 | ABC transporter permease |
| WP_205323020.1 | Molecular chaperone (DnaK) | WP_205319707.1 | Sarcosine oxidase subunit beta family protein |
| WP_205322403.1 | Protein-export chaperone (SecB) | WP_205319708.1 | Sarcosine oxidase subunit delta |
| WP_205323021.1 | Molecular chaperone (DnaJ) | WP_205323396.1 | Sarcosine oxidase subunit alpha family protein |
| WP_205320112.1 | Fe-S protein assembly co-chaperone (HscB) | WP_205323397.1 | Sarcosine oxidase subunit gamma family protein |
| WP_205320116.1 | Fe-S protein assembly chaperone (HscA) | WP_205319702.1 | Choline ABC transporter permease subunit |
| WP_205320454.1 | Molecular chaperone (HtpG) | WP_2053223.1.1 | Peroxiredoxin |
| WP_205319752.1 | Hsp33 family molecular chaperone (HslO) | **Membrane/cell wall alteration**  WP_205322550.1 | Fatty acid desaturase |
| **Oxidative Stress**  WP_205319715.1 | Fe-Mn family superoxide dismutase | **General stress response**  WP_205319547.1, WP_205318997.1, WP_205320944.1 | Universal stress protein |
| WP_205320278.1, WP_205321810.1, WP_205321810.1 | Catalase | WP_205321977.1 | GlsB/YeaQ/YmgE family stress response membrane protein |
| WP_205319028.1 | Glutathione peroxidase | WP_205321423.1 | Peroxide stress protein (YaaA) |
| WP_240162341.1 | Cytochrome c peroxidase | WP_205319633.1 | Serine/threonine protein kinase |
| WP_205321902.1 | Alkyl hydroperoxide reductase | WP_205320534.1 | Transcriptional repressor (LexA) |
| WP_205322028.1 | Organic hydroxide resistance protein |  |  |
| WP_205319315.1 | Thioredoxin TrxA |  |  |
| WP_205320998.1 | Thioredoxin-disulfide reductase |  |  |
| WP_205321215.1 | Thioredoxin family protein |  |  |

**Legends to supplementary figures**

**Fig. S1.** Growth curve of bacteria isolate PAMC28666. Growth was observed in the R2A medium at different NaCl concentrations, pH, and temperature.

**Fig. S3.** Trehalose biosynthetic routes, where green box showing the genes that are possessed by strain PAMC28666 for the synthesis of trehalose using different substrates i.e., *otsA* (trehalose 6-phosphate synthase), *otsB* (trehalose 6-phosphate phosphatase), *treY* (maltooligosyl-trehalose synthase), *treZ* (maltooligosyl-trehalose trehalohydrolase), and *treS* (trehalose synthase).

**Fig. S1**


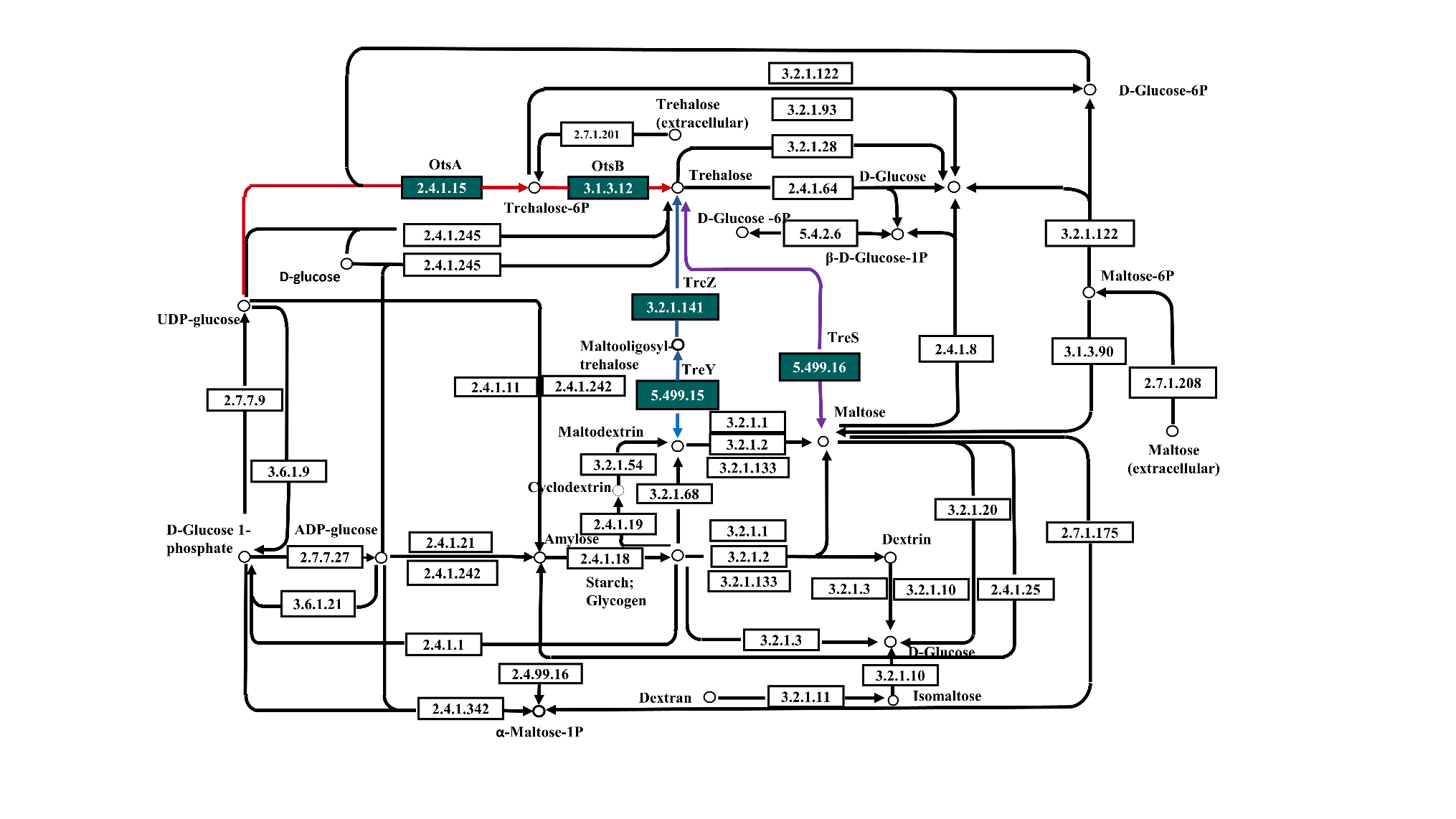


**Fig. S2**
